# The perception and knowledge about episiotomy: A cross-sectional survey involving healthcare workers in a low- and middle-income country

**DOI:** 10.4102/phcfm.v13i1.2424

**Published:** 2021-04-28

**Authors:** Cyprian M. Maphanga, Thinagrin D. Naidoo

**Affiliations:** 1 Department Obstetrics and Gynaecology, Greys Hospital, Pietermaritzburg, South Africa; 2 Nelson R Mandela School of Medicine, University of KwaZulu-Natal, Pietermaritzburg, South Africa

**Keywords:** questionnaire, healthcare workers, episiotomy, obstetricians, professional nurses

## Abstract

**Background:**

Episiotomy was introduced into clinical practice without clear evidence of its benefits.The knowledge and understanding of episiotomy guidelines and practice by healthcare workers is substandard in our setting; hence, the injudicious use of this procedure have led to high rates.

**Aim:**

To assess the knowledge, perception and practice of episiotomy by healthcare workers.

**Setting:**

Research was conducted in a Pietermaritzburg complex, South Africa.

**Methods:**

A questionnaire-based survey was conducted amongst healthcare workers regarding episiotomy practice. In addition to providing demographic data, the participants were requested to respond to 35 proposed statements regarding episiotomy practice. Data were analysed using SPSS (Statistical Package for the Social Sciences) software.

**Results:**

One hundred and forty-two midwives and 66 medical practitioners completed the questionnaires. There were variations in responses to several statements on episiotomy practice by medical practitioners and nurses based on their level of experience. This study found that the majority of HCWs did not have access to a protocol or policy on episiotomy practice in their units; furthermore, nor knowledge of the South African guidelines for maternity care on episiotomy practice. Significantly, more medical practitioners felt a need for more in-service training and an increase in the number of episiotomies performed under supervision. The commonly reported reason for performing an episiotomy by both medical practitioners and midwives was to reduce 3rd – 4th degree perineal tears.

**Conclusion:**

Healthcare workers in our setting displayed poor knowledge about the practice of episiotomy and were not aware of existing national guidelines on episiotomy practice.

## Introduction

Although episiotomy has become one of the most commonly performed procedures in obstetrics, it was introduced into clinical practice without strong scientific evidence of its benefits and hence, it is the subject of much debate.^1^ Episiotomy rates vary widely worldwide, depending on whether the procedure is used restrictively or routinely.^2^ These variations in rates seen worldwide clearly indicate that episiotomy is heavily driven by professional norms, different experiences in training and individual provider preference and not by physiological necessity.^3,4^ These differences could also result from varying personal opinions regarding the benefits of episiotomy and an inconsistency in their acquaintance with the reports from the literature. A Cochrane review has found that a selective episiotomy policy has more benefits compared to routine episiotomy use.^1^ Rates of episiotomies remain high especially in developing countries despite national guidelines uniformly agreeing that restricted use of episiotomy is preferable.^5^ Often, research on episiotomy, including those from low-resource settings, has centred mainly on the views, attitude and experiences of women undergoing episiotomy.^6,7^ Current literature suggests that a policy of restricted episiotomy use is preferable,^8^ but indications for this selective performance are not firmly defined and the benefits are not clearly determined. There is a paucity of data on perception and knowledge about episiotomy involving healthcare workers (HCWs) in low- and middle-income country context. Hence, we decided to conduct this study, with the aim of evaluating the perception and knowledge of episiotomy amongst HCWs in the public health hospitals and clinics with delivery units conducting deliveries in the Pietermaritzburg Metropolitan area of KwaZulu-Natal, South Africa.

## Methodology

This was a cross-sectional observational descriptive survey of HCWs providing intrapartum care at public health facilities in the Pietermaritzburg Metropolitan hospitals (Greys hospital, Edendale hospital and Northdale hospital) and 3 clinics with delivery units with the help of a structured questionnaire on episiotomy practice. The study population was stratified into two groups: medical practitioners (interns, medical officers, registrars and specialist obstetricians) and midwives (advanced and registered).

All the medical practitioners and midwives registered in the department of obstetrics and gynaecology at Greys hospital, Edendale hospital, Northdale hospital and 3 clinics with delivery units volunteered to participate in the study. A survey cover sheet explaining the study was attached to the questionnaire and the participants who gave informed consent went to the next step of questionnaire completion. Subject identifiers were not used in the questionnaire and hence, confidentiality was maintained. A research nurse volunteered to enrol all the willing participants and returned to the principal investigator.

The questionnaires included demographic data including their experience and rankings and statements of episiotomy practice. Midwives and medical practitioners who provided care for women at the time of delivery and had the opportunity to perform episiotomies were eligible to participate.

### Data collection

A pretested and structured self-administered questionnaire was used for data collection. The collected data were checked for completeness and consistency by the principal investigator and supervisor. As per the guidelines, experts in research methodology, obstetrics and gynaecology and oncology further confirmed the validity of the questionnaire before the pilot study. The instrument was pretested on 10 study participants who were working in other health facilities that were not part of the actual study. Findings from the pre-test were used to modify the instrument in terms of clarifying the questions. Minimal changes were required to the survey following pilot testing (e.g. additional options were added to the reasons for episiotomy use), so it was decided that re-piloting was not necessary. The questionnaire was divided into 2 main parts, first dealing with the socio-demographic profile characteristics, professional status of the respondents and knowledge about episiotomy practice. The questionnaire was conducted in English.

A structured questionnaire including 35 statements regarding episiotomy practice was used in this study. Information collected on participant characteristics included profession (medical practitioners or midwife), gender and years of experience in maternity care. The practice questions included the frequency of episiotomy use amongst nulliparous and multiparous women, type of episiotomy used (midline/median, mediolateral or mediolateral) and the reasons for episiotomy use (including the main reason). The questionnaire was divided into two sections. The first section of the questionnaire focused on background demographic data, whilst the second section focused on perception and knowledge about episiotomy. A multiple choice questionnaire was used where participants could choose one or more appropriate answers. Selection of these statements was based on international literature and national guidelines^9,10^ and their validity was evaluated by a feedback from HCWs.

### Statistical analysis

Data were entered into Statistical Package for the Social Sciences (SPSS) version 25 for analysis and a *p* value < 0.05 was considered to be statistically significant. Subgroup analysis was performed for medical practitioners versus midwives and professional experience (≤ 8 years vs. > 8 years). A descriptive statistical analysis of the data was performed using the above software.

### Ethical considerations

Ethical clearance was obtained from the Biomedical Research and Ethics Committee (BREC) of the University of KwaZulu-Natal and the KwaZulu-Natal Provincial Department of Health (BE088/18).

## Results

All 208 obstetricians and midwives who provide delivery care at Greys hospital, Edendale hospital, Northdale hospital and clinics with delivery units were eligible to complete a questionnaire about their perception and knowledge towards episiotomy use. The response rate was 100% (208/208). Two hundred and eight HCWs participated in the study, 66 HCWs (31.7%) were medical practitioners, which included interns, medical officers, registrars and specialist obstetricians, and 142 HCWs (68.3%) were midwives, which included advanced and registered midwives with different durations of experience in obstetrics. Most participants were female with up to 8 years of experience in providing maternity care. Their years of obstetric experience ranged from 0 to 3 years to > 13 years with the majority (31.3%) having 0–3 years of experience (Table 1).

**TABLE 1:** Breakdown of healthcare workers’ health facilities where they were employed, years of experience and training.

Use of episiotomy	*n*	%
**Breakdown of HCWs’ health facilities where they were employed and years of experience**		
**Medical practitioners**		
Interns 1st year	12	5.8
Interns 2nd year	9	4.3
Medical officers > 5 years	13	6.3
Medical officers < 5 years	8	3.8
Registrars 1st 2 years	10	4.8
Registrars 2nd 2 years	5	2.4
Specialist obstetricians	9	4.3
**Midwives**		
Registered midwives	89	42.8
Advanced midwives	53	25.5
**Health facility where registered**		
District hospital	50	29.8
Regional hospital	65	36.1
Tertiary hospital	61	34.1
Clinics	32	
**Years of obstetric experience (years)**		
0–3	65	31.3
4–8	62	29.8
9–12	46	22.1
> 13	35	16.8
**Training of HCWs with regard to episiotomy practice**		
**Have you received any formal training on episiotomy?**		
Yes	160	76.9
No	13	6.3
Unknown	35	16.8
**Where did you get the training?**		
Medical school	76	36.5
Nursing school	83	39.9
During internship	0	0.0
Continued training in labour ward	1	0.5
Special courses	1	0.5
Unknown	48	23.1
**How many episiotomies did you do under supervision?**		
0–5	140	57.3
5–10	65	31.3
> 10	3	1.4
**Do you think there is adequate training with regard to episiotomy practice?**		
Yes	181	87
No	27	12.9
**Do you think there is a need for more in-service training?**		
Yes	94	45.2
No	53	25.5
Unknown	61	29.3
**Does your unit have protocol/policy on episiotomy practice?**		
Yes	49	23.6
No	158	75.9
Don’t know	1	0.5

HCWs, healthcare workers.

A total of 69.7% of the participants, including 30.3% of the medical doctors, defined episiotomy as surgical enlargement of the posterior aspect of the vaginal orifice by an incision to the perineum during the expulsive phase of the 2nd stage of labour. More than 60% (67.3%) of the HCWs had performed at least 5 episiotomies under supervision. The majority (75.9%) of HCWs stated that their obstetric unit did not have a protocol or policy on episiotomy practice. When asked to pinpoint the most important obstacle to reducing episiotomy rates, both medical practitioners and midwives reported lack of training (Table 1 and Table 2).

**TABLE 2:** Health care workers’ perception, knowledge and practice of episiotomy.

Knowledge and perception about episiotomy	*n*	%
**How do you define episiotomy?**		
Surgical enlargement of vaginal orifice during labour	38	18.3
Surgical enlargement of the posterior aspect of the vaginal orifice by an incision to the perineum during last part of 2nd stage of labour	145	69.7
Enlargement of the posterior aspect of the vaginal orifice because of tearing during delivery	5	2.4
All of the above defines an episiotomy	20	9.6
**What anatomical structures are cut during episiotomy?**		
Skin and subcutaneous tissue	50	24.0
Bulbocavernosus muscle and fascia	46	22.1
Transverse perineal muscle	21	10.1
Levator ani muscle and fascia	10	4.8
All of the above	81	38.9
**How many types of episiotomy do you know of?**		
1	46	22.1
3	56	26.9
7	9	4.3
2	97	46.6
**What type of episiotomy is recommended in your facility?**		
Right lateral episiotomy	101	48.6
Left lateral episiotomy	42	20.2
Right mediolateral episiotomy	49	23.6
Left mediolateral episiotomy	14	6.7
Midline episiotomy	2	0.9
**What is the recommended angle from midline for a properly constituted mediolateral episiotomy?**		
40–60	56	26.9
60	16	7.7
< 30	111	53.4
Angle does not matter	27	12.9
**Practice of episiotomy**		
**Do you think that in your practice you need to limit the number of episiotomy?**		
Yes	48	23.1
No	160	76.9
**How often do you perform episiotomy on primigravidae?**		
Always	55	26.4
Sometimes	153	73.6
Rarely	0	0.0
Never	0	0.0
**Do you think episiotomies help expedite deliveries in a busy and overcrowded labour ward?**		
Yes	106	50.9
No	102	49.0
**What do you consider the optimal time to perform episiotomy?**		
When the parturient patient has the urge to push	148	71.2
When 3–4 cm of presenting part visible during contraction	34	16.3
When the perineum is bulging	14	6.7
When delivery is expected with the next 3–4 contractions	6	2.9
When all the above is present	6	2.9
**How often do you take verbal consent for the procedure?**		
Always	14	6.7
Sometimes	171	82.2
Rarely	21	10.1
Never	2	0.9
**How often do you give local anaesthetic before cutting?**		
Always	133	63.9
Sometimes	40	18.2
Rarely	11	5.3
Never	24	11.5

The knowledge of HCWs regarding the episiotomy procedure varied, with only 38.9% being able to identify all the structures that are incised during the procedure. Knowledge about different types of episiotomy was poor, with 48.6% of HCWs responding that the right lateral episiotomy was recommended at their centre. More than 50% of the HCWs stated that less than 30 degree from the midline is the recommended angle for a properly constituted mediolateral episiotomy (Table 2).

Episiotomies were not routine on primigravidae but performed sometimes by 73.6% of the HCWs. More than 50% of the HCWs responded that episiotomies help expedite deliveries in a busy and overcrowded labour ward. The majority (71.2%) of HCWs responded that the parturient’s urge to push was the optimal time to perform episiotomy. The response to taking verbal consent for the procedure varied from never to always, but most HCWs (82.2%) would sometimes take verbal consent with 63.9% of HCWs administering local anaesthetic prior to the procedure. Aiming to reduce 3rd – 4th degree perineal tears was the most commonly identified reason for performing an episiotomy by both medical practitioners and midwives. The second most frequent main reason for performing episiotomies reported by medical practitioners was operative delivery, but this was infrequently reported as a main reason by midwives who do not perform operative deliveries, whilst 64.9% considered a big baby as the foetal indication. The majority (69.7%) of HCWs would give analgesia post-repair of episiotomy, and on discharge, 59.1% would sometimes give prophylactic antibiotics and 50.5% would counsel patients on wound and perineal care post-episiotomy, whilst 71.2% would follow all recommendations stated in the guidelines (Table 3).

**TABLE 3:** Responses to statements of repair and management of episiotomies.

Response to episiotomy practice	*n*	%
**When do you normally repair the episiotomy?**		
Immediately after delivery of the baby	11	5.3
After delivery of the placenta	167	80.3
Depends on the bleeding	30	14.4
**Which suture material do you use to repair episiotomy?**		
Vicryl round 2.0	95	45.7
PDS 3.0	23	11.1
Chromic gut	90	43.3
Vicryl round 1.0	0	0.0
**Which suture technique do you use?**		
Continuous suture	135	64.9
Continuous locking stitches	39	18.8
Interrupted stitches	34	16.3
**What are maternal reasons for performing episiotomy?**		
Primiparity	24	11.5
Perceived tight perineum	87	41.8
To prevent impending perineal tears including 3rd and 4th degree perineal tears	158	75.9
Poor maternal effort	33	15.7
Prolonged 2nd stage of labour	35	16.8
Instrumental deliveries	83	39.9
Previous episiotomy	0	0.0
**What are foetal indications for episiotomy**		
Big baby	135	64.9
Premature babies	31	14.9
Non-reassuring foetal heart tracing to expedite delivery	13	6.3
Breech presentation	48	23.1
Shoulder dystocia	115	55.3
Abnormal positions such as occipito-posterior and face presentations	55	26.4
Multiple pregnancies	7	3.4
**What are the immediate complications of episiotomy**		
Excessive bleeding	148	71.2
Vulva/vaginal hematoma	61	29.3
3rd and 4th degree perineal tears	54	25.9
Infection with abscess formation	38	18.3
Extension of the episiotomy	56	26.9
Deep vaginal lacerations	24	11.5
Wound dehiscence	14	6.7
Rectal injury	41	19.7
**Do you give analgesia post repair of episiotomy and on discharge**		
Always	145	69.7
Sometimes	63	30.3
Rarely	0	0.0
Never	0	0.0
**Do you often give prophylactic antibiotics?**		
Always	62	29.8
Sometimes	123	59.1
Rarely	23	11.1
Never	0	0.0
**How often do you counsel your patients on wound and perineal care?**		
Often	105	50.5
Sometimes	83	39.9
Rarely	23	11.1
Never	0	0.0
**What do you recommend perineal wound care?**		
Use of antiseptic solution after urinating or bowel evacuation	0	0.0
Sit baths	60	28.8
Daily shower and washing with mild soap and water	0	0.0
Stool softeners	0	0.0
All of the above recommended	148	71.2
**What are long-term complications of episiotomies?**		
Dyspareunia	117	56.3
Anal incontinence	47	22.6
Urinary incontinence	20	9.6
Pelvic organ prolapse	15	7.2
Recto-vaginal fistula	86	41.3
Vulvodynia	38	18.3

PDS, P-dioxanone suture.

## Discussion

In this study, we sought to describe the perception knowledge and practice of medical practitioners and midwives regarding episiotomy use in the greater Pietermaritzburg area. We found that medical practitioners and midwives differ with regard to perception and knowledge towards episiotomy. The use of our classification of medical practitioners and midwives makes it possible to distinguish episiotomy practices amongst these HCWs.

There are many different opinions in the literature about using episiotomy restrictively or routinely. The repeated Cochrane collaboration meta-analysis of randomised controlled trials together^1,8^ with the American College of Obstetricians and Gynaecologists,^11^ the National Institute for Health and Care Excellence^12^ and the Swedish guidelines^13^ recommend restrictive rather than routine use of episiotomy. South African national episiotomy guidelines^14^ state that restricted use is preferable. Moreover, one study reported that restrictive use of episiotomy did not only decrease the risks for maternal health but was also less costly than its routine use.^10^ In our study, more than 70.0% of our HCWs did not practice restrictive episiotomy. Routine episiotomy is discouraged according to maternity care guidelines for public health facilities on episiotomy use.^11^ This guideline suggests that episiotomy should only be considered for the following reasons, namely, thick or rigid perineum preventing delivery and prolonging the second stage, foetal distress in the second stage of labour and maternal conditions where rapid delivery is required, for example, cardiac disease, breech or forceps delivery, previous third degree tear and preterm delivery where the perineum is tight.

Whilst the current local and international consensus favours a restrictive episiotomy policy,^14^ our study showed that HCWs are still practising episiotomy routinely regardless of indications and lacked awareness regarding the consequences of episiotomy. This study found that the majority of HCWs did not have access to a protocol or policy on episiotomy practice in their units; furthermore, they had little or no knowledge of the South African guidelines for maternity care on episiotomy practice. These findings were similar to an earlier questionnaire-based study, where accoucheurs lacked awareness of the existing evidence and national guidelines regarding episiotomy use.^15^ However, significantly more midwives compared to medical practitioners were aware of the national guidelines.

In our study, a majority of HCWs felt that there was no need to limit the number of episiotomies. Another recent study reported that 35.4% of the midwives and 44.4% of the obstetricians agreed with the expression ‘that episiotomy should be performed routinely at every birth’.^16^ Furthermore, these authors reported that 37.5% of some midwives and a greater number of medical practitioners agreed that episiotomy gives an opportunity to save more time.^17^ Diniz et al.^17^ observed in their study that a significant number of midwifes in their study felt that episiotomies help expedite deliveries in a busy labour ward and medical practitioners felt that it was easy to repair an episiotomy rather than an irregular large tear.^18^ Furthermore, the authors explain that in a context of shortage of beds in overcrowded hospitals, interventions such as this expedite labour and delivery. Lack of time was a major reason cited by both the midwives and medical practitioners for why they cut the perineum – to deliver women faster.^19^ The same reason was cited in a study on quality of maternity care practices amongst skilled birth attendants in Cambodia: episiotomy was performed in order to accelerate the delivery, given the high number of women in the labour ward.^17^

In our study, 51% of HCWs thought that episiotomy helped expedite deliveries but did not show a preference for suturing episiotomy rather than irregular large tears. Celibi and Guler in their study in 2018 found that the vast majority of HCWs did not see a need to take consent when performing an episiotomy.^15^ We report similar findings.

Earlier studies on the effects of episiotomy do not specify the type of episiotomy and mediolateral episiotomies are mainly preferred in Europe although lateral episiotomies are used in Finland.^18,19,20^ Our findings show that the most common type of episiotomy practised was right lateral episiotomy, and the SA maternity guidelines recommend mediolateral type. In our study, 53.4% of HCWs obtained the angle at which they will perform the episiotomy wrong, with 48.6% performing right lateral episiotomies compared to only 23.6% who were performing right mediolateral episiotomy. It is evident that there is confusion about mediolateral and lateral episiotomies in clinical practice.^21,22^ Additional research comparing mediolateral with lateral episiotomies to avert the confusion in clinical practice is needed. A survey from Nordic countries showed that the majority of obstetricians opted to perform a lateral episiotomy, but 64% called it a mediolateral episiotomy.^23^

Concerns about 3rd – 4th degree tears were both the most commonly reported reason and the primary reason for episiotomy for both medical practitioners and midwives and lack of training in delivering women with an intact perineum was reported as a major obstacle to reducing episiotomy rates.

In our study, there were variations in responses to several statements on episiotomy practice by HCWs based on their level of experience. Significantly, more medical practitioners felt that there was a need for more in-service training and the need to increase the number of episiotomies performed under supervision. Similarly, wide variations in episiotomy practice exist around the world as an expression of the difference in routine episiotomy use between countries and within countries and amongst midwives and obstetricians with the same level of experience in obstetric care.^24,25^

In addition, our study showed that episiotomy indications were subjective, not consistent with international practice guidelines,^20^ variable by country^26,27^ and dependent on the type of obstetrical staff involved.^27^ Also, many of the indications reported by HCWs were not congruent with international clinical guidelines.^20^

The implementation of evidence-based practices remains a significant challenge that requires comprehensive approaches at different levels.^28,29^ As shown by Althabe et al. reducing a common practice such as episiotomy is difficult.^30^

The main limitation of this study was the small number of HCWs included in the study and the relatively small numbers of each subgroup surveyed means that the study was not adequately powered to allow us to be certain that such differences do not exist (a beta error). A larger study would be required to confirm this. Self-assessment does not always reflect well on actual knowledge and ability.

## Conclusion

Healthcare workers in our low- and middle-income setting displayed poor knowledge about the practice of episiotomy and were not aware of the Republic of South Africa (RSA) existing national guidelines on episiotomy practice. The findings in our study are in line with studies carried out in other centres, including those in high-income countries.

## Recommendations

Routine episiotomies are no longer recommended. Still, the procedure is sometimes needed. It is mandatory that HCWs need to be familiar with existing RSA guidelines on episiotomy practice.
